# Reduced internalization of TNF-ɑ/TNFR1 down-regulates caspase dependent phagocytosis induced cell death (PICD) in neonatal monocytes

**DOI:** 10.1371/journal.pone.0182415

**Published:** 2017-08-09

**Authors:** Stephan Dreschers, Christian Gille, Martin Haas, Florence Seubert, Christopher Platen, Thorsten W. Orlikowsky

**Affiliations:** 1 Department of Neonatology, University Children’s Hospital, Aachen, Germany; 2 Department of Neonatology, University Children’s Hospital, Tuebingen, Germany; Columbia University, UNITED STATES

## Abstract

Phagocytosis-induced cell death (PICD) is diminished in cord blood monocytes (CBMO) as compared to cells from adults (PBMO) due to differences in the CD95-pathway. This may support a prolonged pro-inflammatory response with sequels of sustained inflammation as seen in neonatal sepsis. Here we hypothesized that TNF-α mediated induction of apoptosis is impaired in CBMO due to differences in the TNFR1-dependent internalization. Monocytes were infected with *Escherichia coli-*GFP (*E*. *coli-*GFP). Monocyte phenotype, phagocytic activity, induction of apoptosis, and TNF-α/TNF-receptor (TNFR) -expression were analysed. In the course of infection TNF-α-secretion of CBMO was reduced to 40% as compared to PBMO (p<0.05). Neutralization of TNF-α by an αTNF-α antibody reduced apoptotic PICD in PBMO four-fold (p < 0.05 vs. infection with *E*. *coli*). PICD in CBMO was reduced 5-fold compared to PBMO and showed less responsiveness to αTNF-α antibody. CBMO expressed less pro-apoptotic TNFR1, which, after administration of TNF-α or infection with *E*. *coli* was internalized to a lesser extent. With similar phagocytic capacity, reduced TNFR1 internalization in CBMO was accompanied by lower activation of caspase-8 (p < 0.05 vs. PBMO). Stronger caspase-8 activation in PBMO caused more activation of effector caspase-3 and apoptosis (all p < 0.05 vs. PBMO). Our results demonstrate that TNFR1 internalization is critical in mediating PICD in monocytes after infection with *E*.*coli* and is reduced in CBMO.

## Introduction

Pre- and postnatal infections are triggers for a variety of diseases later in life, involving different organ systems. The common link between infection and its secondary sequels, organ damage, evidently is inflammation, and typical diseases of the preterm infant, such as bronchopulmonary dysplasia (BPD) [1], necrotising enterocolitis (NEC) [2], retinopathy of prematurity (ROP) [3], periventricular leucomalacia (PVL) [4], and others have been closely tied to pre- or postnatal infection [5]. Lately, in neuropsychiatric diseases, where one would not necessarily consider perinatal reasons, a crucial role of inflammation during critical phases of brain development has growingly emerged [5].

Inflammatory reactions following infection are mediated by cytokines and effector cells. In addition to granulocytes, monocytes are part of the host’s rapid response component and generate a vigorous antibacterial reaction. The fast and effective elimination of bacteria is the basis for an effective antimicrobial defence and accompanied by cellular and humoral host cell signals. In sepsis of adults, a phenomenon called phagocytosis-induced cell death (PICD) plays a key role in the orchestration of an antibacterial host response, by provoking effector cell apoptosis and thus contributing to a controlled termination of inflammation [6].

PICD is a prerequisite for both, neutralization of bacteria and termination of inflammation. An incomplete elimination of bacteria may result in additional damage to organs and tissues. Attenuation of PICD skews the situation to prolonged cytokine production which may lead to a permanent inflammation resulting in a systemic inflammation response syndrome (SIRS) [7].

The latter clinical picture appears in postnatal sepsis of neonates, as well as in prenatal infection, and is termed fetal inflammatory response syndrome (FIRS). Therefore, effector cell apoptosis is tightly regulated and involves external and internal signalling pathways [8].

In previous studies, we compared phagocytic properties, intracellular degradation and consecutive PICD of neonatal monocytes, obtained from cord blood (CBMO) with those from peripheral blood of healthy adult donors (PBMO). In an *in-vitro*-system using the two most common agents of neonatal sepsis, group B streptococci (GBS) and *Escherichia coli* (*E*. *coli*), CBMO and PBMO showed identical phagocytic and intracellular degradation activities, however CBMO underwent PICD to a lesser extent than PBMO. Inhibition of phagocytosis by Cytochalasin D reduced the induction of PICD. Analyzing factors triggering apoptosis revealed a diminished secretion of CD95L, one of the death-ligands of the TNF-family, in CBMO [9].We went on to demonstrate, that apoptosis occurs in phagocyting and non-phagocyting PBMO [10].

TNF-α is a pleiotropic cytokine mediating cell proliferation, inflammation and cell death [11]. The pro-inflammatory signalling is initialized by ligating both receptors TNFR1 and TNFR2 at the plasma-membrane. TNF-α is synthesized as a membrane-anchored protein, which can be cleaved by metalloproteases. The cleaved, soluble TNF-α predominantly binds to TNFR1, whereas membrane anchored TNF-α (also designated as pro-TNF-α) or mTNF-α has higher affinity to TNFR2 [12]. Uptake of ligand bound TNFR1 was found to be a prerequisite of pro-apoptotic signalling [13].

Activation of apoptosis inducing caspases in response to extracellular signals is designated the extrinsic pathway of apoptosis. TNFR1 internalization activates caspase-8 and -3 by engaging TRADD, RIP-1 and FADD, thereby forming a signalosome engaged in the extrinsic pathway [13].

Previous results suggested that apoptosis of PBMO after infection with *E*. *coli* occurred via internalization of TNFR1, and indicated a relevant role for TNF-α [10].

Regulation of intracellular signalling can be achieved by cleavage of either ligands or receptors, respectively. This process often designated as shedding can critically reduce ligands/receptors on the cell surface and thereby down-regulate the transmission of a signal. Here we tested the hypothesis, that reduced apoptosis of CBMO during phagocytosis of *E*. *coli* was mediated by altered TNF-α/TNFR signalling.

## Material and methods

### Patients

The study protocol was approved by the Ethics Committees of Aachen University Hospital (Permission No: EK150/09, Oct. 6, 2009, signed by Profs G. Schmalzing and U. Buell, respectively). All adult participants involved gave written consent to use their blood samples. All term neonates were delivered spontaneously and did not exhibit signs of infection, as defined by clinical status, which was controlled by trained neonatologists repeatedly until discharge, white blood cell count, Interleukin-6 (IL-6) and C-reactive protein. Mothers with amnion infection, prolonged labour (> 12 hours), small for gestational infants (SGA) and preterm infants before 36 weeks of gestation were excluded. Umbilical cord blood was placed in heparin-coated tubes (4 IE/ml blood), immediately following cord ligation as described before [14].

### Bacteria

#### *E*. *coli*-GFP

*E*. *coli* DH5α, an encapsulated K12 laboratory strain, carrying the green fluorescent protein (*gfp*)-mut2 gene (*E*. *coli*-GFP) was a generous gift from Prof. Dr. Dehio (University of Basel, Switzerland) and was used for phagocytosis as previously described. Bacteria were freshly grown in Lennox-L-Broth-medium (Thermo Fisher Scientific, Waltham, MA, USA) until early logarithmic growth, resuspended in PBS and used immediately. Infection was performed at a multiplicity of infection (MOI) of 25:1 which was achieved by dilution with PBS. The phagocytosis assays were performed as described [12]. During the infection interval, culture medium without antibiotics was used. The phagocytosis index was calculated (CD14+GFP+ monocytes: CD14+ monocytes) and analysed by flow cytometry.

#### Reagents

Antibodies to CD14 (MɸP9; MEM18), TNFR1 (55R-286), TNFR2 (hTNFR1-M1) and Ig-matched controls (IgG1, IgG2b) were from BD Biosciences and Immunotools (Heidelberg, Germany and Friesoythe, Germany, respectively). Antibodies binding to cleaved caspase-3 and -8 were purchased from Cell Signaling (clone 9661, New England Biolabs, Danvers, MA, USA) and Thermo Fisher Scientific (clone MA5-15054, Waltham, MA, USA), respectively. Staining was performed according to the manufacturer`s recommendations. After removal of primary antibodies, a secondary appropriate fluorochrome-labelled antibody was used. Propidium iodide (PI), isopropyl-β-D-thiogalactopyranoside (IPTG) and antibiotics were purchased from Sigma (Munich, Germany). The TUNEL apoptosis kit was purchased from Roche (Mannheim, Germany). Metalloproteinase inhibitor GM6001 (Chemicon International, Darmstadt, Germany) was added 1h prior to infection to a final concentration of 0.01mM. The anti-TNF-α antibody (αTNF-α mAb), a chimeric molecule combining the ligand-binding domain of the TNF-receptor 2 and the Fc-domain of human IgG1 (ENBREL, Pfizer–Wyeth, Hamburg, Germany) was added ad a final concentration of 1μg/ml simultaneously with infection. The CD95L blocking antibody (ZB4) was purchased from ENZO (Lörrach, Germany) and used as described previously. In brief, 4μg/ml ZB4 was added to the cultured cells prior to infection [9]. In experiments aimed to investigate the synergistic effect of TNF-α and CD95L, 0.5μg/ml αTNF-α and 2μg/ml of ZB4 were used.

The TNFR2 inhibiting antibody (MAB-726, R&D systems, Minneapolis, MN, USA) was used at a final concentration of 0.75μg/ml and added 30 min prior to infection. Antibodies for immunoblot detection of RIP, TRADD, actin and secondary HRP-coupled antibodies were form Santa Cruz Biotechnologies (Santa Cruz, CA, USA).

TNF-α was purchased from eBiosciences (eBiosciences-Natutec, Frankfurt, Germany), aliquoted freshly after dilution in PBS and used in apoptosis induction assays in final concentrations titrated up to of 5ng/ml.

The caspase-8 inhibitor Z-Isoleucine-Glutamine-Threonine-Asparagine-fluormethyl ketone (Z-IETD-FMK, purchased by PromoKine, Heidelberg, Germany) was administered 1 h prior to infection in a final concentration of 2μM. The pan-caspase inhibitor zVAD-fmk (G7238) was purchased from Promega (Promega Corp., Madison, WI, USA) at a final concentration of 50μM 1 hour before infection.

#### Mononuclear cell cultures

Peripheral blood mononuclear cells from adults and cord blood mononuclear cells (PBMC and CBMC) were isolated by density gradient centrifugation on Ficoll cushions (Amersham, Chalfont St. Giles, UK) as described previously [14]. Washed cells were resuspended in VLE RPMI-1640 (Biochrom, Germany). For analysis of post-phagocytic reactions, cells were counted in an ultraplane Neubauer hemocytometer, placed at 2x10^6^ cells/ml in flat bottom 24 well cell culture plates (Greiner, Solingen, Germany), containing 10% heat-inactivated fetal calf serum (FCS, Biochrom, Germany) and gentamycin (Sigma, Munich, Germany, 200μg/ml), and were incubated at 37°C.

To compare post-phagocytic reactions in whole blood and leucocytes, whole blood (50 μl) was stained with anti-CD14 antibodies for 15 min on ice. Via FACS analysis the percentage of monocytes was determined and the appropriate volume of whole blood subjected to the *in- vitro*-infection-assay.

#### Flow cytometry

A daily calibrated FACS-Canto flow cytometer (Becton Dickinson, Mountain View, NJ, USA) was used to perform phenotypic analysis. To prevent nonspecific binding, cells were incubated with 10% fetal calf serum on ice for 10 minutes before staining with pacific-blue (PB)-, fluorescein-isothiocyanate (FITC)-, phycoerythrin (PE)-, allophycocyanin (APC)-, or isotype-specific immunoglobulin-labelled monoclonal antibodies for 20 minutes over ice in the dark. Monocytes were gated by forward (FSC), side scatter (SSC), and CD14 expression.

TNFR1 internalization assays utilizing anti-TNFR antibodies were performed as described before [10]. Biotin-labelled TNF-α for monitoring internalization was from eBiosciences (Frankfurt, Germany). In brief, 1x106 mononuclear cells were resuspended in PBS and Biotin-labelled TNF-α added in a final concentration as recommended by the manufacturer. Mononuclear cells were kept for the indicated time interval under standard culture conditions, before streptavidin-FITC was administered. After washing in PBS specimen were subjected to FACS analysis.

### Apoptosis assays

#### Annexin-V assay

Annexin V was provided by Immunotools (Friesoythe, Germany). 2μl of annexin V solution was added to 10^6^ cells in CaCl_2_ supplemented PBS (2.5 mmol final concentration) for 20 min on ice in the dark as described by the manufacturer.

#### TUNEL assay

Cells were stained with CD14 mAb for 15 minutes at RT before fixation in paraformaldehyde (2% v/v in PBS). Subsequent steps of the TUNEL assay were performed according to the manufacturer`s recommendations (Roche, Mannheim, Germany). Fixed, permeabilized and DNAse I treated mononuclear cells served as positive controls.

#### Detection of hypodiploid nuclei

DNA fragmentation was assessed according to Nicoletti and previously described [10]. In brief, washed cells were slowly resuspended in 2ml of -20°C ethanol 70% with continuous vortexing and stored for four hours at -20°C. Cells were washed twice, resuspended in 50μl PBS containing 13 units RNAse (DNAse free; Sigma, Germany) and incubated for 15 minutes at 37°C. 180μl of PI (70μg/ml) was added, incubated for 20 minutes and analysis was performed immediately. Alternatively, mononuclear cells were stained with CD14 antibody for 15 minutes at RT to identify monocytes. A fixation with paraformaldehyde (2% v/v in PBS) for 2 hrs at RT replaced the ethanol fixation. Afterwards, cells were permeabilized by incubation in PBS-T (PBS, Triton X-100 0,1% v/v) for 20 minutes at RT, washed twice in PBS, resuspended in PBS-PI (PBS, 70μg/ml PI and 13 units RNAse) and incubated for 10 minutes at RT before analysis by flow cytometry. Cell-doublets were discriminated by assessment of PI-width/PI-area as described [15]. For further dissection of apoptotic and non-apoptotic DNA degradation, living cells were incubated with Vybrant dye (Thermo Fisher Scientific, Waltham, MA, USA) according to the manufacturer`s recommendations. FACS analysis allows detection of sub-G1 populations representing apoptotic cells. Cells in G1 phase which were also positive for PI were designated as cells undergoing non-apoptotic cell death. The latter method was used to determine TNF-α concentrations inducing apoptotic cell death only. We found that TNF-α concentration below 5 ng/ml did not induce apoptosis. A TNF-α concentration higher than 5 ng/ml induced also non-apoptotic cell death and was not suitable for this study.

#### ELISA

The TNF-α enzyme-linked immunosorbent assay (ELISA) was purchased from Ebiosciences (Ebiosciences-Natutec, Frankfurt, Germany) and used according to the manufacturer`s recommendations. The read-out was executed in a spectra max 340PC ELISA reader (molecular devices, Sunnyvale, CA, USA) with a sensitivity from 4–500pg/ml.

#### Immunoblot

For the immunoblot analysis 6x10^6^ cells were washed twice with PBS and lysed in Laemmli-lysis buffer. After heating to 96 C ^o^ for 5 min contaminating DNA was destroyed by pipetting samples through a syringe (gauge 23) before they were subjected to gel electrophoresis (SDS-PAGE). SDS-PAGE was performed according to standard protocols. Antibodies were diluted according to the manufacturers`recommendations. For imaging a LAS 3000 (Fujifilm, Düsseldorf, Germany) was used. Signal strength was quantified with the multi-gauge software (Fujifilm, Düsseldorf, Germany).

### Statistical analysis

Results are expressed as mean +/- standard deviation. Error bars represent standard deviations. Values of p < 0.05 were considered significant. Analyses were done with statistical software (performing student`s t-test, one-way and two-way ANOVA adjusted according to Bonferroni-Holm for multiple group comparisons as provided by GraphPad Software Statistical Package, La Jolla, CA,USA).

Data distribution was assessed by a Shapiro-Wilk normality test.

## Results

### *E*.*coli* induces reduced TNF-α secretion in CBMO which is functional in PICD

We infected monocytes with *E*.*coli*-GFP for four hours and analysed their phagocytic activity. As shown before [16], phagocytic indices did not differ between PBMO and CBMO (Panel A in S1 Fig). Phagocytic indices were similar after infection in whole blood and leukocyte fractions, indicating that periphagocytic reactions *in-vitro* are comparable to the *ex-vivo* situation (Panel A in S1 Fig).

**Fig 1:**
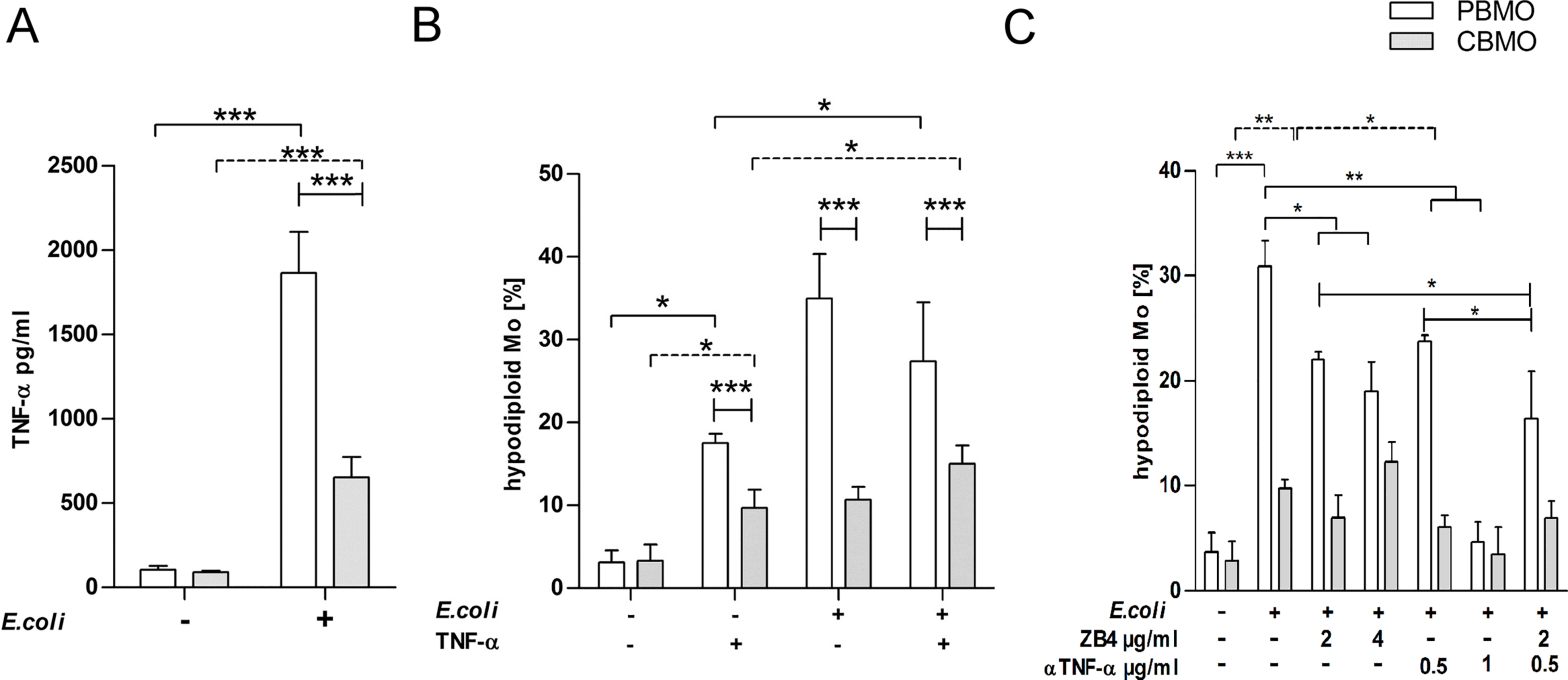
*E*. *coli* induces TNF-α secretion which is involved in induction of apoptosis in PBMO and CBMO. Assessment of secreted TNF-α 4 h p.i. from supernatants of PBMO and CBMO (A, n = 7; ***p < 0.005, clamped bars, student`s t-test; ***p < 0.005, blunt-ended bars, two-way ANOVA). Induction of apoptosis 4 h p.i. assessed by Nicoletti assay in CD14+ monocytes after indicated treatment (B, n = 8; *p < 0.05, clamped bars, student`s t-test; blunt-ended bars, two-way ANOVA). PICD assay as shown in (B), but with addition of blocking antibodies ZB4 and αTNF-α (final concentration given below the chart) as indicated (C, n = 3; *p < 0.05, clamped bars, student`s t-test; blunt-ended bars, two-way ANOVA).

Infection with *E*.*coli* for four hours raised the concentration of soluble TNF-α up to 2 ng/ml in supernatants of infected PBMO. Soluble TNF-α from infected CBMO was approximately 2.5-fold lower (Fig 1A, p < 0.05). The TNF-α secretion in infected whole blood samples was comparable to that found after preparation of PBMO and CBMO (Panel B in S1 Fig).

To test if TNF-α caused apoptosis in CBMO and PBMO we added TNF-α into the cultures. We observed a significant induction of apoptosis in both groups. However, the apoptosis rate of PBMO was 3.7 times higher than CBMO (Fig 1B). Whereas the combination of TNF-α and infection with *E*.*coli* did not increase apoptotic rates in PBMO, it in contrast significantly enhanced the CBMO apoptosis rates, compared to infection alone (p < 0.05, Fig 1B).

Employing the *E*.*coli*-GFP *in-vitro* infection model, we assessed the induction of PICD by three independent methods and observed reduced apoptotic rates in CBMO (Fig 1B and 1C and Table 1). PICD- induced apoptosis was stronger in PBMO compared to CBMO.

**Table 1:** Assessment of apoptosis in monocytes.

	PBMO						CBMO					
	Annexin		TUNEL		hypodiploid DNA		Annexin		TUNEL		hypodiploid DNA	
	Mean	SD	Mean	SD	Mean	SD	Mean	SD	Mean	SD	Mean	SD
**Non-infected**	**1.75**	2.9	**2.2**	1.6	**3.1**	1.1	**1.7**	0.5	**2.3**	0.9	**0.8**	3.2
***E*.*coli* infected**	47.6^**#^	6.1	32.5^**#^	6.2	35^**#^	5.3	7.4^**#^	2	6.6^**^	1.2	**10.7**^#**^	2.3

Monocytes were infected with *E*.*coli-*GFP for 4 hours and free bacteria were removed. Apoptosis was detected by annexin V to detect phosphatidylserine in the outer leaflet of the plasmamembrane (n = 7), which is an initial phase of apoptosis. Hypodiploid nuclei were detected by utilizing the method of Nicoletti et al. (see material and methods)n = 6).DNA strand breaks, which occur in the late phase of apoptosis were detected by the TUNEL assay ((n = 12), all assays, ** p < 0.01 non-infected vs. infected (# p < 0.005 ANOVA PBMO vs. CBMO)).

Cells were infected with *E*. *coli*-GFP for 4 hours with an MOI of 25.

To shed light on the question, whether PICD is caused predominantly by CD95L/CD95 or by TNF-α/TNFR1 signalling, we added αTNF-α and CD95L neutralizing antibody ZB4 in two concentrations to infected monocytes (Fig 1C). Addition of the lower concentration of either αTNF-α or ZB4 reduced PICD to 70% (Fig 1C, compare 2^nd^, 3^rd^ and 5^th^ columns). Combination of both blocking antibodies had a synergistic effect and reduced PICD up to 50% (Fig 1C, compare 2^nd^ and 7^th^ columns). However, addition of the twofold concentration of ZB4 did not further reduce PICD whereas twofold concentration of αTNF-α resulted in a complete blockage of PICD (Fig 1C, compare 2^nd^, and 6^th^ columns).

The results indicate that induction of PICD is not restricted to CD95L/CD95 signalling. This effect could not be observed for CBMO, were ZB4 failed to reduce PICD (Fig 1C, 2^nd^ vs. 3^rd^ column). The αTNF-α antibody in turn could also block the already reduced PICD in infected CBMO.

As shown for phagocytic indices and TNF-α secretion, apoptosis was induced in adult and neonatal whole blood to comparable extent to that observed for PBMO and CBMO (Fig 1 and Panel C in S1 Fig).

### Diminished TNFR1 expression and -internalization after infection leads to reduced apoptosis in CBMO

In a recent publication we demonstrated the effect of TNFR1 internalization with regard to pro-apoptotic signalling [10]. Therefore, it seemed reasonable to compare this process in PBMO and CBMO.

**Fig 2:**
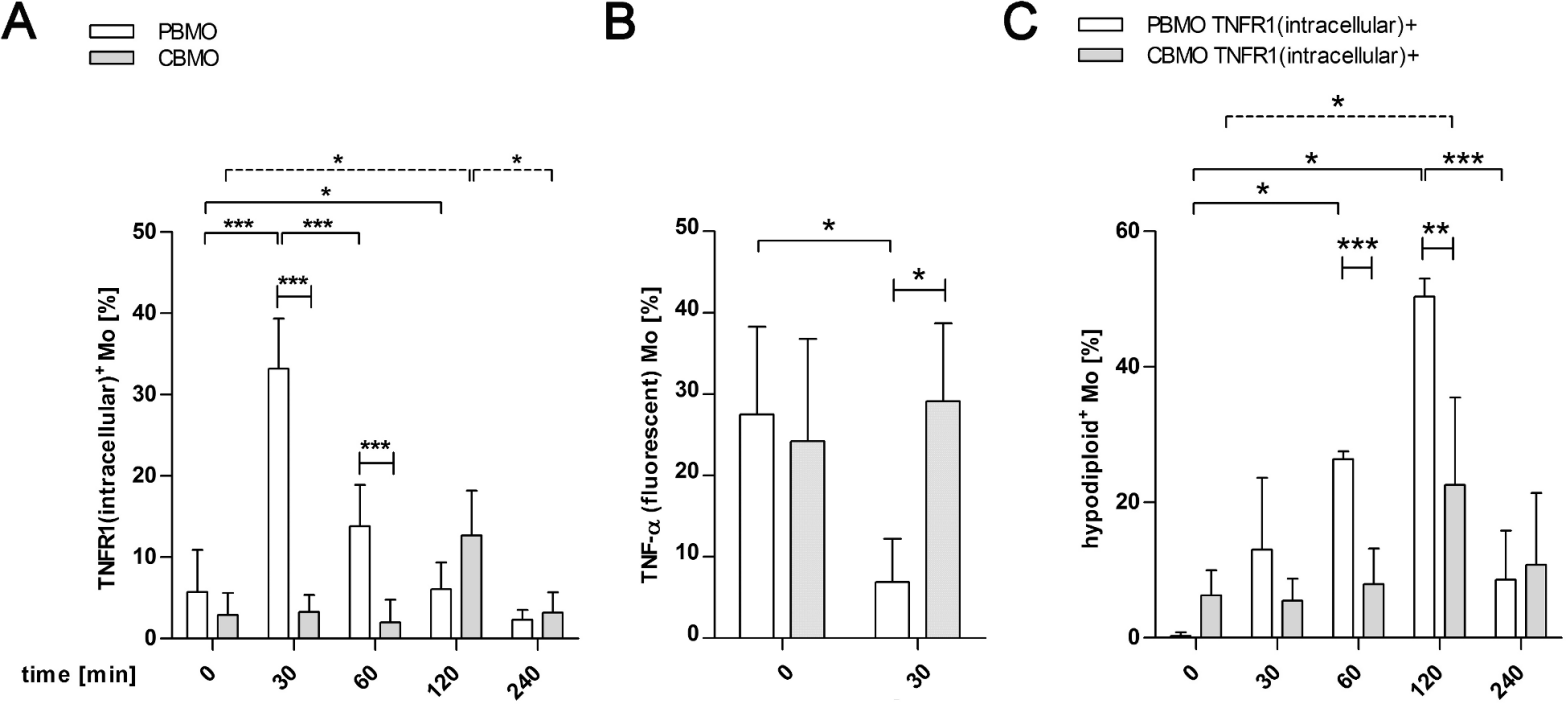
TNFR1 dependent internalization of TNF-α causes apoptosis. Assessment of internalized TNFR1 in PBMO and CBMO after indicated intervals (A, n = 8; *p < 0.05, ***p < 0.005, clamped bars, student`s t-test; ***p < 0.005, blunt-ended bars, two-way ANOVA). Detection of TNF-α on the plasma-membrane of monocytes (B, n = 3; p < 0.05; clamped bars, student`s t-test; blunt-ended bars, two-way ANOVA). Apoptosis induced by addition of TNF-α was measured in monocytes exhibiting internalized TNFR1 (C, n = 7, *p < 0.05, **p < 0.01, ***p < 0.005, clamped bars, student`s t-test; blunt-ended bars, two-way ANOVA).

Tracking of TNFR1 internalization after addition of TNF-α showed a rapid uptake of TNF-α /TNFR1 in PBMO, but a delay in CBMO. Over a period of 240 min, CBMO exhibited significant TNFR1 internalization only at 120 min p.i. which dropped again at 240 min p.i. (Fig 2A). We further tracked biotinylated TNF-α on monocytes and, in contrast to PBMO, observed TNF-α retention on the plasma-membrane of CBMO (Fig 2B).

The PBMO that internalized TNFR1 after addition of TNF-α showed a significant induction of apoptosis from 60 min to 120 min p.i. with a peak 120 min p.i. (Fig 2C). In contrast, the percentage of TNFR1 internalizing CBMO undergoing apoptosis was diminished compared to PBMO (Fig 2C).

Additionally, we determined induction of apoptosis in PBMO and CBMO which did not internalize TNFR1. No significant amounts of apoptotic monocytes could be detected (S2 Fig).

### Altering the TNFR1 surface expression can pilot PICD in monocytes

As shown in Fig 1, phagocytosis of *E*.*coli* induced a stronger pro-apoptotic potential compared to TNF-α stimulation. Reduction of surface TNFR1 expression could reflect internalization or shedding of the receptor. We addressed the question whether cycling of TNFR1 from the surface to the cytoplasm during *E*.*coli* infection has an influence on the induction of PICD.

**Fig 3:**
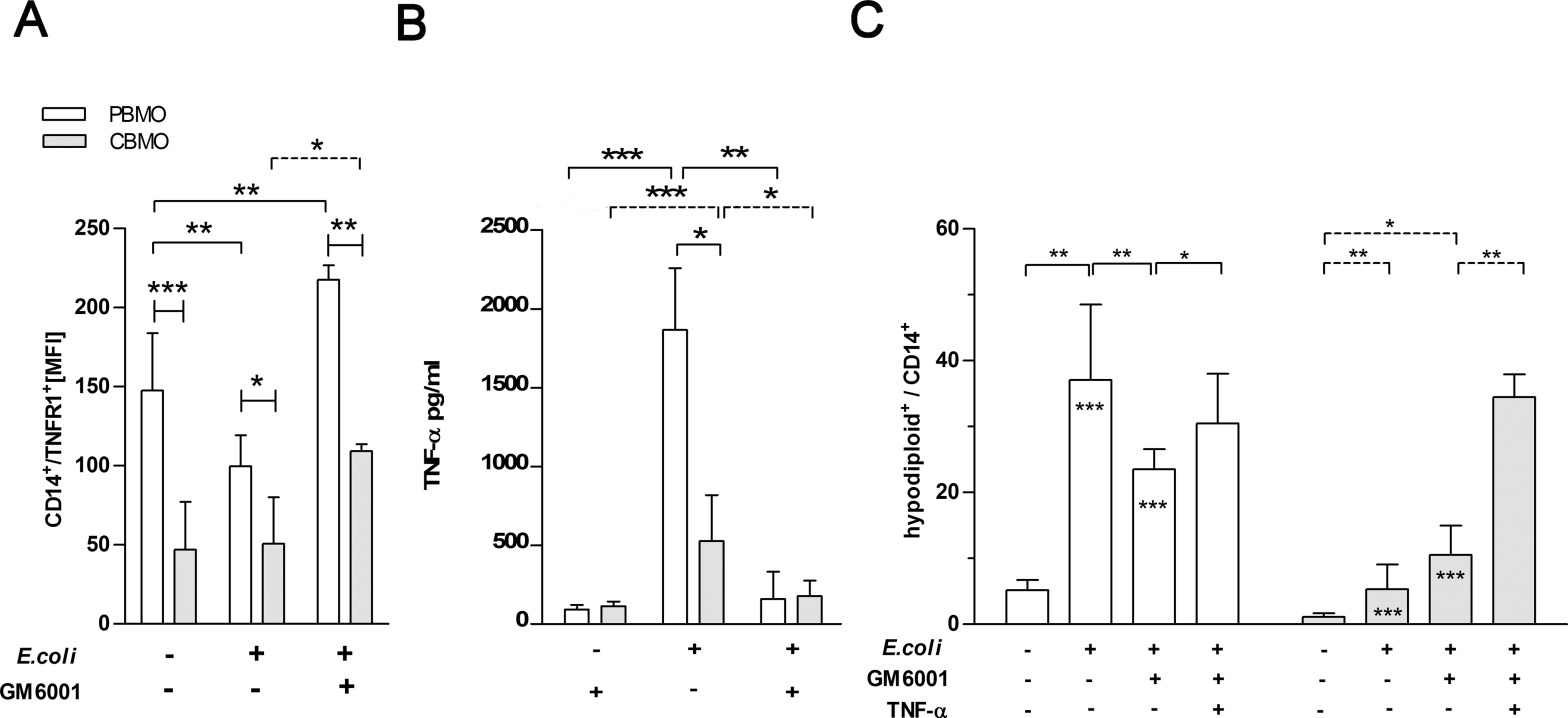
TNFR1 expression after infection is functional in induction of monocytic apoptosis. TNFR1 expression on the plasma-membrane of PBMO and CBMO before and after infection depicted as mean expression of TNFR1 on all monocytes (A, n = 6; *p < 0.05, **p < 0.01, ***p < 0.005, clamped bars, student`s t-test; blunt-ended bars, two-way ANOVA). To groups indicated, the metalloprotease inhibitor GM6001 was added. TNF-α secretion is diminished by GM6001 (B, n = 3; *p < 0.05, **p < 0.01, ***p < 0.005, clamped bars, student`s t-test). Detection of apoptotic PBMO and CBMO after infection and/or addition of GM6001 and TNF-α (C, n = 8, *p < 0.05, **p < 0.01, clamped bars, student`s t-test *within bars, p < 0.005, two-way ANOVA).

In the non-infected groups 2.5-fold more PBMO expressed TNFR1 compared to CBMO (13.6 ± 3.9% vs. 3.7 ± 1.2%; p < 0.005). In the infected groups PBMO significantly down-regulated TNFR1, whereas TNFR1 densities in CBMO remained equal on the cell surface (p < 0.05; Fig 3A, second columns). The TNFR1-densities [MFI] were lower on CBMO (TNFR1 32.8 ± 28.8) than on PBMO (TNFR1 137.8 ± 30, Fig 3A; p < 0.05).

In order to prevent the TNFR1 from being shed from the surface [17], we administered the metalloprotease inhibitor GM6001 to infected and non-infected monocytes. The infection-induced down-regulation of TNFR1 on PBMO could be entirely blocked by GM6001; on CBMO we found higher densities than on infected monocytes only (Fig 3A, 3^rd^ columns).

Due to the fact that GM6001 could block shedding of TNFR1 and/or shedding of its ligand TNF-α resulting in lower soluble TNF-α (Fig 3B) which is a prerequisite for internalization and induction of apoptosis [13], we added TNF-α into the cultures. To further evaluate the role of TNFR1 expression in pro-apoptotic signalling, we compared the induction of apoptosis in infected PBMO and CBMO in the absence or presence of GM6001 (Fig 3C). Addition of GM6001 to infected monocytes increased TNFR1 surface expression and led to lower apoptotic rates in PBMO but not in CBMO. Infected and GM6001-treated CBMO showed apoptosis rates similar to infected PBMO (Fig 3C).

Reduced TNF-α dependent apoptosis of CBMO could be due to amplified binding to TNFR2 which we found equally expressed in PBMO and CBMO (S1 Table). Pre-incubation with a blocking TNFR2 antibody did not interfere with the induction of apoptosis in infected PBMO and CBMO (S2 Table).

### *E*. *coli* induced TNFR1-internalization leads to activation of caspase-8 and effector caspase-3

**Fig 4:**
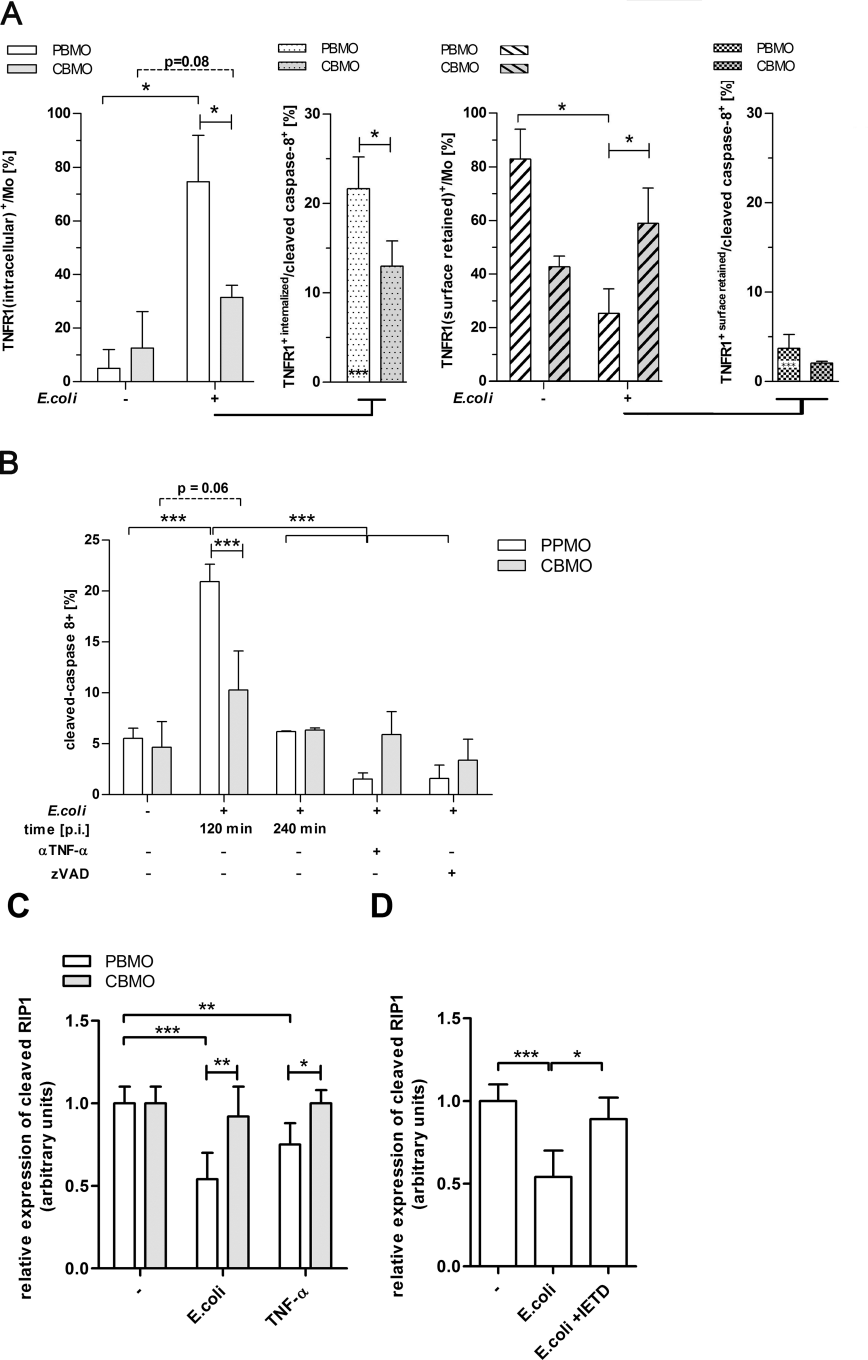
Internalization of TNFR1 induces caspase-8 and -3 cleavage after *E*.*coli* infection. Assessment of TNFR1 internalization in monocytes 2 hrs p.i. (A; n = 11, left panel) and proteolytic cleavage of caspases-8 in monocytes with internalized TNFR1 (A; n = 3,panel with dotted bars;*p < 0.05, ***p < 0.005, clamped bars, student`s t-test; blunt-ended bars, one- and two-way ANOVA). Monocytes which exhibited surface retained TNFR1 (A, panel to the right, hatched bars) were also tested for cleaved caspase-8 (second right panel, both, *p < 0.05, ***p < 0.005, clamped bars, student`s t-test; blunt-ended bars, one- and two-way ANOVA).The percentage of monocytes expressing cleaved caspase-8 is given (B). Pre-treatment with either zVAD or αTNF-α antibody as described (n = 10, *p < 0.05, ***p < 0.005, clamped bars, student`s t-test; blunt-ended bars, two-way ANOVA, brunched clamped bars, one-way ANOVA). The cleavage of RIP was monitored by calculating the quotient of RIP/RIPc after quantification of RIP and RIPc signals. If RIPc outweighs RIP the quotient is less than 1 which is the value found in non-treated probes (C; n = 3; *p < 0.05, **p < 0.01, ***p < 0.005, clamped bars, student`s t-test, blunt-ended bars, two-way ANOVA).

To further characterize the downstream events of TNFR1-signalling, we tested, whether internalization of TNFR1 leads to autoproteolytic activation of caspase-8 (Fig 4A). Infected monocytes were analysed for TNFR1 internalization (Fig 4A, left). In monocytes with internalized TNFR1, as compared to cells which had not internalized their TNFR1, the cleavage of caspase-8 was significantly more prominent in PBMO (p < 0.05 vs. CBMO; Fig 4A right panel), suggesting that infection-induced internalization of TNFR1 initiates caspase-8 activation.

After infection with *E*. *coli* some groups received an αTNF-α mAb, a pancaspase inhibitor (zVAD; Fig 4B), or the specific caspase-8 inhibitor (IETD; Fig 4C). The latter was given in order to demonstrate the dependency of caspase-3 induction on caspase-8 activation.

Infection with *E*. *coli* led to increased cleavage of caspase-8 in PBMO (p<0.01; Fig 4B), while this effect was strongly reduced in CBMO 120 min p.i. Caspase-8 activation was time-limited, because 240 min p.i., both, PBMO and CBMO did not show caspase-8 activation significant different to the non-infected controls (Fig 4B). Addition of αTNF-α mAb to infected monocytes significantly reduced activities of caspase-8 and caspase-3 in PBMO and CBMO (Fig 4B and 4C, 3^rd^ columns).

The pancaspase inhibitor zVAD inhibited caspase-8 activation on PBMO, but, had no additional inhibiting effect (Fig 4B). Cleavage of caspase-3 in infected monocytes (Fig 4C) was inhibited by the specific caspase-8 inhibitor IETD (p < 0.05, infected groups vs. infected groups treated with IETD), suggesting that caspase-3 cleavage in this process was dependent on caspase-8. In infected CBMO, cleaved caspase-3 activation was lower (p < 0.05 vs. PBMO), but also greatly diminished by IETD.

Additionally, we checked whether TRADD and RIP; proteins forming a signalling complex engaging caspase-8, were expressed differentially in PBMO and CBMO. Both TRADD and RIP were expressed at comparable levels in PBMO and CBMO either in non-treated and infected or TNF-α treated monocytes (Panels A and B in S3 Fig). However, in *E*.*coli*-infected PBMO RIP was processed to the smaller cleaved form RIPc (Fig 4C). The same was found for TNF-α administration. Significant, *E*.*coli* or TNF-α induced RIPc formation could not be found in CBMO and was diminished in PBMO, treated with caspase-8 inhibitor IETD (Fig 4D).

**Fig 5:**
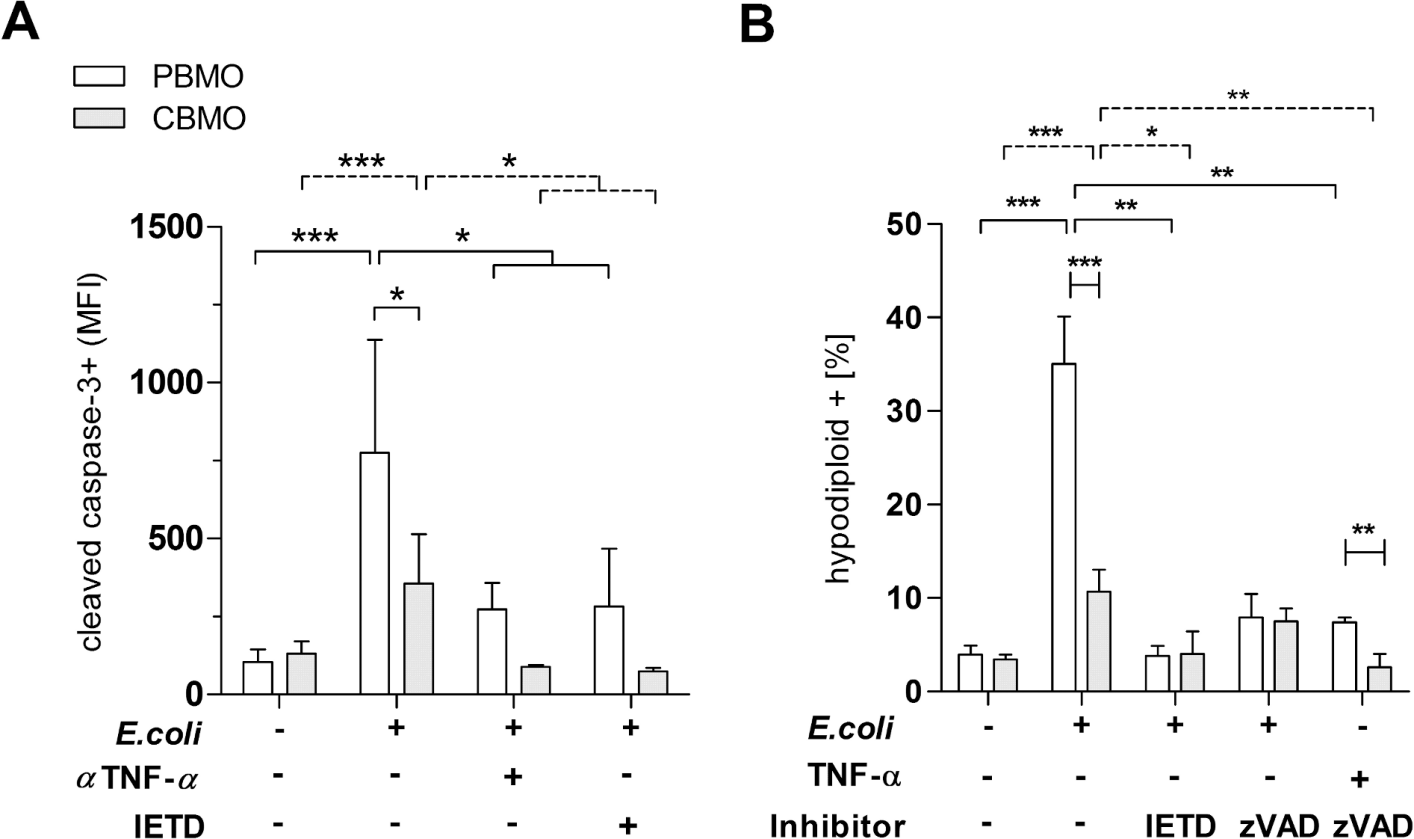
Inhibition of caspase-8 leads to reduction of cleaved caspase-3 and mounts for diminished PICD. Detection of cleaved caspase-3 4 h p.i. Groups which received the α-TNF-α mAb,andIETD were indicated (A; n = 10, *p < 0.05, ***p < 0.005, clamped bars, student`s t-test; blunt-ended bars, two-way ANOVA). Detection of hypodiploid nuclei after indicated treatment and administration of caspase inhibitors 4 h p.i. (B; n = 10, *p < 0.05, **p < 0.01 ***p < 0.005, clamped bars, student`s t-test; blunt-ended bars, two-way ANOVA).

Next, we addressed the question whether we could inhibit PICD by blocking TNF-α-mediated caspase-8 signalling in monocytes. Therefore we added the caspase-8 inhibitor IETD to infected monocytes (Fig 5). Compared to the group of infected monocytes without IETD treatment, IETD blocked the induction of apoptosis in PBMO and CBMO, suggesting that in both, PBMO and CBMO caspase-8 activation is a prerequisite for the induction of apoptosis after infection (Fig 5, 2^nd^ vs. 3^rd^ column; p < 0.05).

## Discussion

The present study compared the induction of PICD in CBMO and PBMO utilizing an *in-vitro E*.*coli* infection model. In contrast to PBMO, reduced PICD in CBMO was accompanied by low TNF-α secretion and diminished TNFR1 internalization. Downstream of TNF/TNFR1 internalization caspase-8 and consecutively caspase-3 activation was reduced in CBMO, leading to their diminished apoptosis.

Reduced levels of TNF-α in neonatal monocytes after infection or challenge with bacteria (Fig 1A) or bacterial PAMPs (such as LPS) are a matter of debate with most studies indicating a bias towards reduced TH1 cytokine production in CBMO. In response to most TLR ligands, most reports found neonatal innate immune cells, including monocytes, conventional and plasmacytoid dendritic cells producing less IL-12p70 and IFN-ɣ, moderately less TNF-α but as much or even more IL-1ß, IL-6, IL-23, and IL-10 than adult cells [18].In concurrence with our study, reduced extracellular TNF-α levels in *E*. *coli-*infected purified monocytes have been described [19] [20–22]. Dampening inflammation is thought to be essential for the fetal immune system to avoid rejection by the mother and termination of pregnancy [23]. Therefore reduced secretion of TNF-α could be beneficial for the fetal environment, albeit bearing the risk of reduced capacity to fight of infection and of prolonged activation of immune effector cells by due to missing pro-apoptotic signaling. In this regard, the isolation of PBMO and CBMO via depletion of granulocytes and removal of serum factors could be limiting factors of our model. However, comparison of our published results to the whole blood assays (S1 Fig), showed that basic results, such as phagocytic activity, TNF-α secretion and induction of apoptosis were similar in both experimental setups.

We found that the susceptibility towards PICD of infected CBMO increased, when exogenous TNF-α was added (Fig 1B, 2^nd^ vs. 4^th^ columns). These data, together with the observation that PICD of PBMO, but not CBMO could be greatly diminished by blocking TNF-α (Fig 1C and Table 1) pointed towards a crucial role of the TNF-α pathway in these processes. Addition of exogenous TNF-α led to rapid internalization of TNFR1 in PBMO but not CBMO (Fig 2A). Since apoptosis mainly occurred in TNFR-1-internalizing monocytes, the difference in TNFR-1 internalization could account for the difference in apoptosis observed between PMBO and CBMO (Fig 2C). This is in line with the observation that CBMO expressed lower densities of TNFR1 on the surface of infected cells (Fig 3A, 1^st^ vs. 2^nd^ column) and that TNF-α remained on the surface of CBMO (Fig 2B). Downstream signalling of internalized TNF-α is regulated by the recruitment of adaptor proteins like TRADD and RIP forming two competing protein complexes driving either apoptosis or pro-inflammatory NFkappaB activation [13, 24–26]. TRADD can recruit caspase-8 which cleaves RIP1 [27]. This truncated RIPc contains the death domain enabling the binding of FADD. In turn, the anti-apoptotic activation of the NFkappaB pathway is reduced. Since blockage of caspase-8 by IETD (Fig 5) abrogates apoptosis it is feasible to argue that reduced TNFR1 internalization in CBMO causes reduced apoptosis. Furthermore, we observed cleavage of RIP in infected or TNF-α treated PBMO (Fig 4B and 4C). This process was caspase-8 dependent and could not be observed in CBMO (Fig 4B and 4C). The biological significance of the rather modest effect of TNF-α on *E*.*coli*-induced PICD in cord blood (CBMO) remains to be elucidated; although the administration of anti-TNF-α antibody inhibited PICD of CBMO significantly (Fig 1C).

Like TNF-α, CD95L of which reduced production in CBMO was recently published to cause diminished apoptosis [9], recruits an adaptor protein FADD [24]. CD95L activated FADD can directly engage caspase-8 and, unlike internalized TNF-α/TNFR1, does not require further signals. This is in line with our findings that supplementation with bioactive CD95L increased the level of endogenous CD95L in CBMO and enhances apoptosis to levels observed in PBMO [9].

CBMO which are challenged by both *E*.*coli* and TNF-α died more frequently compared to infected only or TNF-α treated only CBMO (Fig 1B). This minor but significant effect, which is lacking in PBMO cannot be attributed to higher expression of RIP and TRADD in CBMO *per se*, because these proteins were shown to be equally expressed (Panels A and B in S3 Fig). Therefore, processing of RIP and activation of caspase-8 could be a consequence of TNFR1 internalization, which is reduced in CBMO. RIP However, some methodical limitations cannot rule out a coincidence of the observed processes. Future experiments should be conducted to purify internalized TNFR1 signalosomes which can be tested for caspase and RIP activation. As pointed out before TNF-α/TNFR1 ligation is potentially an inducer of an apoptosis modulating signal cascade. TNF-α secretion could induce CD95L expression suggesting that CD95L/CD95 signalling is a predominant pro-apoptotic pathway without requirement of TNFR1 internalization. Evidence for phagocytosis-triggered activation of the pro-apoptotic cascade involving caspases -8 and -3, was described for *Staphylococcus aureus* [28]. Phagocytosis also regulates the expression of small Bcl-2 like proteins [23]. For the BCL-2 like protein t-Bid a connecting function to the extrinsic and intrinsic apoptosis cascade could be shown [29], defining caspase-8 as a hinge in an auto-activating loop accelerating apoptosis.

In this regard, numerous bacterial pathogens, among them *E*.*coli*, *Salmonella* and *Shigella* produce and secrete effector proteins which impair the signalling machinery of TNF-family ligands and their receptors by inhibition of downstream mediators such as FADD, TRADD and caspases [24, 30].

In previous publications we found caspases-8 and -9 activated after infection with GFP-*E*.*coli* [31]. Caspase-8 and -9 are functional in the intrinsic- and extrinsic apoptosis pathway and in turn activate effector caspases, e.g. caspase-3.

In infected PBMO cleaved caspase-8 was found at significantly higher levels than in CBMO (Fig 4A, left). Furthermore, caspase-8 activation in PBMO was markedly inhibited by an αTNF-α antibody (Fig 4B, 3^rd^ vs. 2^nd^ columns), indicating that TNF-α initiates the downstream activation of effector caspase-3 (Fig 4C, 3^rd^ vs. 2^nd^ columns) via caspase-8 (Fig 4C, 4^th^ columns), leading to apoptosis (Fig 5).

In this manuscript, we also addressed the question, whether shedding of TNF-α and its receptor TNFR1 interferes with pro-apoptotic signalling. Blockage with the metalloprotease-inhibitor GM6001 (Fig 3) resulted in increased densities of TNFR1 on infected monocytes (Fig 3A 2^nd^ vs. 3^rd^ columns), and decreased the number of infected apoptotic PBMO (Fig 3C, 2^nd^ vs. 3^rd^ columns). Adding TNF-α into this scenario (Fig 3C, 4^th^ columns) had no synergistic effect on the PICD of PBMO, but led to a significant increase of PICD of CBMO to the extent as comparable to infected PBMO.

Besides TNF-α/TNFR1/TNFR2 and CD95L/CD95 other ligands/receptors may play a role in regulation of apoptosis in PBMO and CBMO. TRAIL receptors can be activated and also activate the extrinsic caspase signalling pathway. The TRAIL receptor expression was not found reduced in CBMO compared to maternal PBMO [32], but expression of TRAIL ligand has not been examined in detail yet. To date, the model of sustained inflammation is favored to explain fatal outcome of neonatal sepsis. However, our data emphasizes the possibility that the reduced capacity of the neonatal immune system could contribute to pathology. These monocytes could have harmful effects on cells in the surrounding tissue, by inducing further apoptosis and tissue damage as it was shown for the lung [33]. To what extent this may influence the course of neonatal sepsis, remains to be elucidated *in vivo*.

## Supporting information

S1 Fig Periphagocytic reactions are similar in *ex-vivo* and *in-vitro* assays. Whole blood cells of adults (white bars) and neonates (grey bars) and MNC were infected with *E*.*coli* (MOI 25) and the PI (A, calculated as CD14+GFP+ monocytes: CD14+ monocytes, see the material and method section) and the TNF-α secretion (B) measured. Induction of apoptosis in non-infected and infected whole blood of adults (white hatched bars) and neonatal (grey hatched bars) samples was also determined (n = 3, **p < 0.01 ***p < 0.005, clamped bars, student`s t-test; blunt-ended bars, two-way ANOVA).
(TIF)

S2 Fig Surface retention of TNFR1 does not induce apoptosis. Percentage of hypodiploid nuclei after addition of TNF-α was measured in monocytes exhibiting TNFR1 on the surface (compare Fig 2C, n = 5).
(TIF)

S3 Fig RIP and TRADD protein expression. Quantification of immunoblots comparing the expression of TRADD (A) and RIP (B) in PBMO and CBMO under indicated treatment (n = 3; MOI 25, 2 h p.i.; TNF-α, 5 ng/ml 2 h post treatment). Representative immunoblots are shown below the charts. (C–D) One of three immunoblots comparing RIP (designated as RIP) cleavage in PBMO and CBMO under indicated treatment (C) and RIP cleavage in *E*.*coli* infected PBMO with or without IETD treatment (compare lane 2 and 3). Note, that non-treated PBMO show different expression of cleaved RIP due to varied exposure time.
(TIF)

S1 Table TNFR2 expression in PBMO and CBMO. TNFR2 expression on PBMO and CBMO was assessed by FACS staining (n = 3, given are mean values and standard deviation).
(XLSX)

S2 Table Blocking of TNFR2 receptor does not interfere with apoptosis. PBMO and CBMO received inhibiting TNFR2 antibody when indicated. Apoptosis was assessed by detection of hypodiplid nuclei and is given as percentage of apoptotic cells (n = 4).
(XLSX)

## Abbreviations

α: anti
CBMC: cord blood mononuclear cells
CBMO: CD14-positive peripheral blood monocytes from cord blood
CD95L, CD95: cell death promoting factor (FAS-L) and its receptor (FAS)
*E.coli*: *Escherichia* *coli*
GFP: green fluorescent protein
LPS: Lipopolysaccharide
MNC: mononuclear cells
MOI: multiplicity of infection
MFI: mean fluorescence index
PAMPs: Pathogen associated molecular patterns
PBMC: peripheral blood mononuclear cells
PBMO: CD14-positive peripheral blood monocytes from adults
PBS: phosphate buffered saline
PBS-T: phosphate buffered saline Triton-X 100 supplemented
PICD: phagocytosis induced cell death
PI: propidium iodide
p.i.: post infection
RIP(c): receptor-interacting-protein 1 (cleaved)
RT: room temperature
TUNEL: terminal UTP-deoxynucleotidyl-transferase
TNFR1: Tumor necrosis factor-α receptor 1
TNFR2: Tumor necrosis factor-α receptor 2
TRADD: tumor-necrosis-factor-receptor-type-1-associated-DEATH-domain-protein
v/v: volume per volume
WBC: whole blood cells

## References

[1] Hentschel J, Berger TM, Tschopp A, Muller M, Adams M, Bucher HU, et al (2005) Population-based study of bronchopulmonary dysplasia in very low birth weight infants in Switzerland. Eur J Pediatr 164: 292–297. doi: 10.1007/s00431-005-1623-1 15711958 10.1007/s00431-005-1623-1

[2] Bracci R, Buonocore G (2003) Chorioamnionitis: a risk factor for fetal and neonatal morbidity. Biol Neonate 83: 85–96. 12576751 10.1159/000067956

[3] Hauspurg AK, Allred EN, Vanderveen DK, Chen M, Bednarek FJ, Cole C, et al (2011) Blood gases and retinopathy of prematurity: the ELGAN Study. Neonatology 99: 104–111. doi: 10.1159/000308454 20689332 10.1159/000308454 PMC2939988

[4] Kohelet D, Shochat R, Lusky A, Reichman B (2006) Risk factors for seizures in very low birthweight infants with periventricular leukomalacia. J Child Neurol 21: 965–970. doi: 10.1177/08830738060210111301 17092463 10.1177/08830738060210111301

[5] Gantert M, Been JV, Gavilanes AW, Garnier Y, Zimmermann LJ, Kramer BW (2010) Chorioamnionitis: a multiorgan disease of the fetus? J Perinatol 30 Suppl: S21–30. doi: 10.1038/jp.2010.96 20877404 10.1038/jp.2010.96

[6] Hotchkiss RS, Coopersmith CM, McDunn JE, Ferguson TA (2009) The sepsis seesaw: tilting toward immunosuppression. Nat Med 15: 496–497. doi: 10.1038/nm0509-496 19424209 10.1038/nm0509-496 PMC3786779

[7] Serhan CN, Savill J (2005) Resolution of inflammation: the beginning programs the end. Nat Immunol 6: 1191–1197. doi: 10.1038/ni1276 16369558 10.1038/ni1276

[8] Hotchkiss RS, Nicholson DW (2006) Apoptosis and caspases regulate death and inflammation in sepsis. Nat Rev Immunol 6: 813–822. doi: 10.1038/nri1943 17039247 10.1038/nri1943

[9] Gille C, Dreschers S, Leiber A, Lepiorz F, Krusch M, Grosse-Opphoff J, et al (2013) The CD95/CD95L pathway is involved in phagocytosis-induced cell death of monocytes and may account for sustained inflammation in neonates. Pediatr Res 73: 402–408. doi: 10.1038/pr.2012.196 23269121 10.1038/pr.2012.196

[10] Dreschers S, Gille C, Haas M, Grosse-Ophoff J, Schneider M, Leiber A, et al (2013) Infection-induced bystander-apoptosis of monocytes is TNF-alpha-mediated. PLoS One 8: e53589 doi: 10.1371/journal.pone.0053589 23349721 10.1371/journal.pone.0053589 PMC3547953

[11] Tchikov V, Bertsch U, Fritsch J, Edelmann B, Schutze S (2011) Subcellular compartmentalization of TNF receptor-1 and CD95 signaling pathways. Eur J Cell Biol 90: 467–475. doi: 10.1016/j.ejcb.2010.11.002 21144616 10.1016/j.ejcb.2010.11.002

[12] Richter C, Messerschmidt S, Holeiter G, Tepperink J, Osswald S, Zappe A, et al (2012) The tumor necrosis factor receptor stalk regions define responsiveness to soluble versus membrane-bound ligand. Mol Cell Biol 32: 2515–2529. doi: 10.1128/MCB.06458-11 22547679 10.1128/MCB.06458-11 PMC3434479

[13] Schneider-Brachert W, Tchikov V, Neumeyer J, Jakob M, Winoto-Morbach S, Held-Feindt J, et al (2004) Compartmentalization of TNF receptor 1 signaling: internalized TNF receptosomes as death signaling vesicles. Immunity 21: 415–428. doi: 10.1016/j.immuni.2004.08.017 15357952 10.1016/j.immuni.2004.08.017

[14] Gille C, Spring B, Tewes LJ, Loffler J, Dannecker GE, Hoffmann MK, et al (2006) Diminished response to interleukin-10 and reduced antibody-dependent cellular cytotoxicity of cord blood monocyte-derived macrophages. Pediatr Res 60: 152–157. doi: 10.1203/01.pdr.0000228345.58509.7b 16864695 10.1203/01.pdr.0000228345.58509.7b

[15] Wersto RP, Chrest FJ, Leary JF, Morris C, Stetler-Stevenson MA, Gabrielson E (2001) Doublet discrimination in DNA cell-cycle analysis. Cytometry 46: 296–306. 11746105 10.1002/cyto.1171

[16] Gille C, Leiber A, Mundle I, Spring B, Abele H, Spellerberg B, et al (2009) Phagocytosis and postphagocytic reaction of cord blood and adult blood monocyte after infection with green fluorescent protein-labeled Escherichia coli and group B Streptococci. Cytometry B Clin Cytom 76: 271–284. doi: 10.1002/cyto.b.20474 19288547 10.1002/cyto.b.20474

[17] Scheller J, Chalaris A, Garbers C, Rose-John S (2011) ADAM17: a molecular switch to control inflammation and tissue regeneration. Trends Immunol 32: 380–387. doi: 10.1016/j.it.2011.05.005 21752713 10.1016/j.it.2011.05.005

[18] Kollmann TR, Crabtree J, Rein-Weston A, Blimkie D, Thommai F, Wang XY, et al (2009) Neonatal innate TLR-mediated responses are distinct from those of adults. J Immunol 183: 7150–7160. doi: 10.4049/jimmunol.0901481 19917677 10.4049/jimmunol.0901481 PMC4556237

[19] Angelone DF, Wessels MR, Coughlin M, Suter EE, Valentini P, Kalish LA, et al (2006) Innate immunity of the human newborn is polarized toward a high ratio of IL-6/TNF-alpha production in vitro and in vivo. Pediatr Res 60: 205–209. doi: 10.1203/01.pdr.0000228319.10481.ea 16864705 10.1203/01.pdr.0000228319.10481.ea

[20] Liu CA, Wang CL, Wang FS, Huang HC, Chuang H, Chen RF, et al (2005) Higher spontaneous and TNFalpha-induced apoptosis of neonatal blood granulocytes. Pediatr Res 58: 132–137. doi: 10.1203/01.PDR.0000163396.89508.5C 15879291 10.1203/01.PDR.0000163396.89508.5C

[21] Pedraza-Sanchez S, Hise AG, Ramachandra L, Arechavaleta-Velasco F, King CL (2013) Reduced frequency of a CD14+ CD16+ monocyte subset with high Toll-like receptor 4 expression in cord blood compared to adult blood contributes to lipopolysaccharide hyporesponsiveness in newborns. Clin Vaccine Immunol 20: 962–971. doi: 10.1128/CVI.00609-12 23595503 10.1128/CVI.00609-12 PMC3697450

[22] Levy O, Coughlin M, Cronstein BN, Roy RM, Desai A, Wessels MR (2006) The adenosine system selectively inhibits TLR-mediated TNF-alpha production in the human newborn. J Immunol 177: 1956–1966. 16849509 10.4049/jimmunol.177.3.1956 PMC2881468

[23] Leiber A, Graf B, Spring B, Rudner J, Kostlin N, Orlikowsky TW, et al (2014) Neonatal monocytes express antiapoptotic pattern of Bcl-2 proteins and show diminished apoptosis upon infection with Escherichia coli. Pediatr Res 76: 142–149. doi: 10.1038/pr.2014.74 24850312 10.1038/pr.2014.74

[24] Jin Z, El-Deiry WS (2006) Distinct signaling pathways in TRAIL- versus tumor necrosis factor-induced apoptosis. Mol Cell Biol 26: 8136–8148. doi: 10.1128/MCB.00257-06 16940186 10.1128/MCB.00257-06 PMC1636728

[25] Micheau O, Tschopp J (2003) Induction of TNF receptor I-mediated apoptosis via two sequential signaling complexes. Cell 114: 181–190. 12887920 10.1016/s0092-8674(03)00521-x

[26] Schutze S, Schneider-Brachert W (2009) Impact of TNF-R1 and CD95 internalization on apoptotic and antiapoptotic signaling. Results Probl Cell Differ 49: 63–85. doi: 10.1007/400_2008_23 19132322 10.1007/400_2008_23

[27] Lin Y, Devin A, Rodriguez Y, Liu ZG (1999) Cleavage of the death domain kinase RIP by caspase-8 prompts TNF-induced apoptosis. Genes Dev 13: 2514–2526. 10521396 10.1101/gad.13.19.2514 PMC317073

[28] Baran J, Weglarczyk K, Mysiak M, Guzik K, Ernst M, Flad HD, et al (2001) Fas (CD95)-Fas ligand interactions are responsible for monocyte apoptosis occurring as a result of phagocytosis and killing of Staphylococcus aureus. Infect Immun 69: 1287–1297. doi: 10.1128/IAI.69.3.1287-1297.2001 11179290 10.1128/IAI.69.3.1287-1297.2001 PMC98019

[29] Kaufmann T, Strasser A, Jost PJ (2012) Fas death receptor signalling: roles of Bid and XIAP. Cell Death Differ 19: 42–50. doi: 10.1038/cdd.2011.121 21959933 10.1038/cdd.2011.121 PMC3252833

[30] Giogha C, Lung TW, Pearson JS, Hartland EL (2014) Inhibition of death receptor signaling by bacterial gut pathogens. Cytokine Growth Factor Rev 25: 235–243. doi: 10.1016/j.cytogfr.2013.12.012 24440054 10.1016/j.cytogfr.2013.12.012

[31] Gille C, Leiber A, Spring B, Kempf VA, Loeffler J, Poets CF, et al (2008) Diminished phagocytosis-induced cell death (PICD) in neonatal monocytes upon infection with Escherichia coli. Pediatr Res 63: 33–38. doi: 10.1203/PDR.0b013e31815b8e9f 18043500 10.1203/PDR.0b013e31815b8e9f

[32] Zauli G, Monasta L, Vecchi Brumatti L, Agnoletto C, Volpi P, Secchiero P, et al (2013) The circulating levels of TRAIL are extremely low after delivery but rapidly recover in both mothers and newborns. Cytokine 64: 51–53. doi: 10.1016/j.cyto.2013.05.005 23722000 10.1016/j.cyto.2013.05.005

[33] Behnia M, Robertson KA, Martin WJ, 2nd (2000) Lung infections: role of apoptosis in host defense and pathogenesis of disease. Chest 117: 1771–1777. 10858414 10.1378/chest.117.6.1771

