# Supplementary figures and images for: Reduced internalization of TNF-ɑ/TNFR1 down-regulates caspase dependent phagocytosis induced cell death (PICD) in neonatal monocytes
Source: PLoS One. 2017 Aug 9;12(8):e0182415. doi: 10.1371/journal.pone.0182415 (PMC5549969; doi:10.1371/journal.pone.0182415)

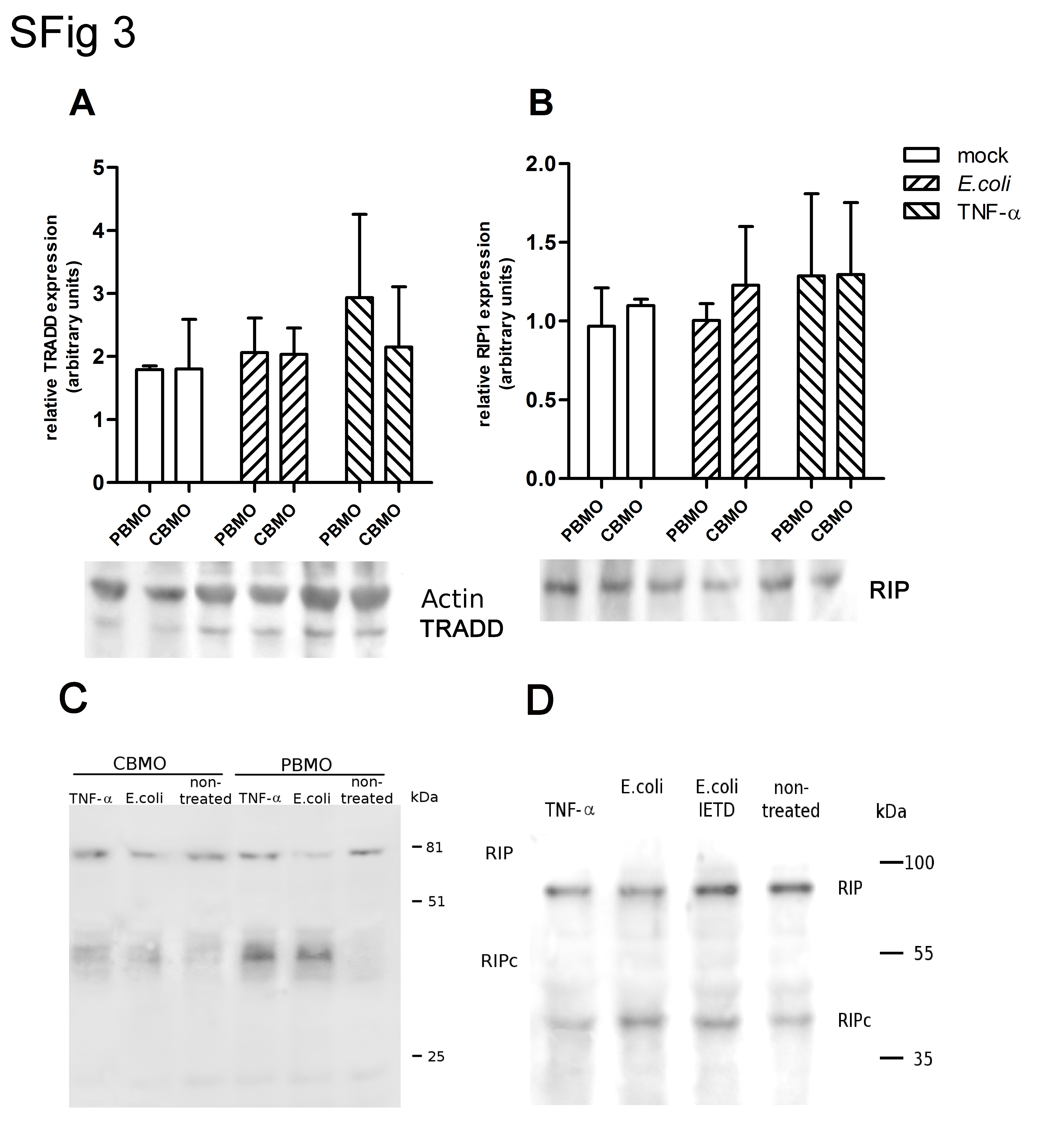

Supplement: S3 Fig — Quantification of immunoblots comparing the expression of TRADD (A) and RIP (B) in PBMO and CBMO under indicated treatment (n = 3; MOI 25, 2 h p.i.; TNF-α, 5 ng/ml 2 h post treatment). Representative immunoblots are shown below the charts. (C–D) One of three immunoblots comparing RIP (designated as RIP) cleavage in PBMO and CBMO under indicated treatment (C) and RIP cleavage in E.coli infected PBMO with or without IETD treatment (compare lane 2 and 3). Note, that non-treated PBMO show different expression of cleaved RIP due to varied exposure time. (TIF) [file pone.0182415.s003.tif]
